# Incidence and predictors of mortality among neonates admitted with perinatal asphyxia at west Oromia tertiary hospitals, Ethiopia, 2022

**DOI:** 10.1186/s12887-023-04313-6

**Published:** 2023-09-19

**Authors:** Dawit Tesfaye Daka, Chalachew Adugna Wubneh, Tewodros Getaneh Alemu, Bewuketu Terefe

**Affiliations:** 1 Department of Pediatrics and Neonatal Nursing, School of Nursing and Midwifery, Institute of Health Sciences, Wollega University, Nekemte, Ethiopia; 2 Department of Pediatrics and Child Health Nursing, School of Nursing, College of Medicine and Health Sciences, University of Gondar, Gondar, Ethiopia; 3 Department of Community Health Nursing, School of Nursing, College of Medicine and Health Sciences, University of Gondar, Gondar, Ethiopia

**Keywords:** Perinatal asphyxia, Incidence, Predictors, Mortality, Ethiopia

## Abstract

**Background:**

Perinatal asphyxia is one of the preventable and treatable causes of neonatal mortality. However, it is the fifth-largest cause of under-five mortality. Even with management advancements, it remains one of the key public health issues in underdeveloped countries, including Ethiopia. Comorbidities are also understated; therefore, adequate information regarding the incidence of death and its predictors is required.

**Methods:**

A four-year retrospective follow-up study was conducted from October 3 to November 2, 2022. From a total sample size, of 655, 616 data were collected by nurse through follow-up reviews charts using Kobo Toolbox software. The data was exported to STATA Version 14 for analysis. The Cox proportional hazard assumption was checked, and the model for the data was selected using Akaike Information Criteria. Finally, an adjusted hazard ratio with 95% CI was computed, and variables with a P-value < 0.05 in the multivariable analysis were taken as significant predictors of death.

**Result:**

The overall incidence of mortality was 38.86/1000 (95% CI: 33.85–44.60). The median time of follow-up was 15 days (95% CI: 14–20). The proportion of deaths was 202 (32.79%, 95% CI: 29.18–36.61) among neonates with perinatal asphyxia. While the distance from health facility > 10 km is (AHR: 2.25; 95% CI: 1.60–3.17), direct oxygen (AHR: 1.83; 95% CI: 1.35–2.48), APGAR score (Appearance, Pulse, Grimace, Activity, and Respiration) < 3 at the fifth minute (AHR: 2.63; 95% CI: 1.03–6.73), prolonged rupture of membrane (AHR: 1.41; 95% CI: 1.02–1.94), and stage III hypoxic ischemic encephalopathy (AHR: 2.02; 95% CI: 1.18–3.47) were predictors of mortality among neonates with perinatal asphyxia.

**Conclusion:**

According to this study’s findings, high neonatal mortality due to perinatal asphyxia requires proper intervention regarding membrane rupture, APGAR score (Appearance, Pulse, Grimace, Activity, and Respiration), oxygen use, stage III hypoxic-ischemic encephalopathy, and residence distance.

## Background

Perinatal asphyxia (PNA) is the failure of a neonate to establish and sustain normal respiration after birth [1]. Perinatal asphyxia causes progressive hypoxemia, hypercarbia, and metabolic acidosis, as well as multi-organ failure [1, 2]. Perinatal asphyxia is an injury to the fetus or neonate caused by inadequate oxygenation (hypoxia) or inadequate blood flow (ischemia) to numerous organs and systems, including the central nervous system, cardiovascular system, respiratory system, and hematologic system [3, 4].

Globally, 2.4 million infant deaths occurred in the first month of life in 2020 [5]. Neonatal mortality declined less quickly between 1990 and 2020 than under-five mortality, and the highest neonatal mortality rate in 2020 was on the African continent, with 27 deaths per 1000 live births, and perinatal asphyxia was the major cause of neonatal death on this continent [6, 7]. After pneumonia, diarrhea, neonatal infections, and preterm birth problems, perinatal asphyxia ranks fifth in terms of the causes of under-five infant mortality, and it accounts for about 23% of neonatal deaths [8, 9]. Nearly 60% of all neonatal deaths occur in the first three days of life, and the majority of preterm, malformation, and asphyxia-related deaths occur in the first week [10]. Perinatal asphyxia rates are higher in developing countries, ranging from 4.6 to 1000 in Cape Town to 7–26 per 1000 in Nigeria, and mortality rates may be as high as 40%, with the precise burden of severe neurological disability being higher in underdeveloped countries [5, 11, 12].

Low birth weight, preterm deliveries, malpresentation, mode of delivery, meconium-stained amniotic fluid, chorioamnionitis, and prolonged rupture of membranes were shown to be risk factors for poor outcomes for neonates who were born asphyxiated [13, 14]. Numerous clinical, pathological, biochemical, and metabolic alterations result from perinatal hypoxia, which increases the risk of death [15]. The survival status of neonates with birth asphyxia was low, particularly in the primary care facilities in Ethiopia [16]. According to research, stage III birth asphyxia is the leading cause of perinatal and neonatal death [17].

Despite improvements in the management of perinatal care, ANC follow-up strategies, and the accessibility of NICU (neonatal intensive care unit) care, perinatal asphyxia continues to account for the majority of neonatal intensive care unit (NICU) admissions and is one of the leading causes of neonatal deaths in underdeveloped countries [18–20]. A study conducted in Vietnam identified that prematurity, asphyxia, and designated congenital malformations are underestimated because most of the research did not consider the comorbidities [21, 22]. A retrospective cohort study establishes perinatal asphyxia as a substantial factor in illness and mortality among neonates, and the incidence of mortality from PNA was not adequately reported, but it is high according to some studies, particularly in developing countries [13, 23, 24]. In Ethiopian healthcare institutions, midwives’ and nurses’ retention of their knowledge of neonatal resuscitation practices could contribute to an increase in the number of neonates dying from asphyxia [25].

The neonatal mortality rate is still high and linked to some predictors, and the leading cause of death was perinatal asphyxia [26]. Neonatal mortality is unexpectedly growing in Ethiopia, while it has been stagnating in many low-income countries at 28–33 per 1000 live births [27]. Perinatal asphyxia was the main reason for admission (55.3%), which increases the economic impact and the increased rate of neonate death, according to a facility-based cross-sectional study, but lacks the incidences of death and related factors among neonates admitted with perinatal asphyxia [28, 29]. However, perinatal asphyxia is acknowledged as the greatest contributor to neonatal mortality, but previous research in this field has focused on the prevalence of PNA and missed some important variables. The goal of this study is to determine the incidence of PNA-related mortality and identify the factors that predict mortality in neonates with perinatal asphyxia.

## Methods

### Study design and period

Institution-based retrospective follow-up study design was employed from October 3 to November 2, 2022.

### Study setting

The study was conducted at five tertiary hospitals in the west Oromia region. Those hospitals were Mettu Karl Referral Hospital (MKRH), Jimma University Medical Centre (JUMC), Wollega University Referral Hospital (WURH), Nekemte Specialized Hospital (NSH), and Ambo University Referral Hospital (AURH). Each hospital is far from Addis Ababa, the Ethiopian capital, at 600 km, 420 km, 325 km, and 125 km, respectively. All of them provide tertiary NICU service when there are 10 nurses (4 neonatal nurses), 1 pediatrician at MKRH, 11 nurses (2 neonatal nurses), and 2 pediatricians at NSRH, 22 nurses (8 neonatal nurses), 8 pediatricians, and 35 residents at JUMC. Eleven nurses (6 neonatal nurses), 5 pediatricians, and 13 residents at WURH, and 14 nurses (0 neonatal nurses), 7 pediatricians, and 12 residents at AURH are actively working in each hospital. Average annual ANC services were 6100 in WURH, 7500, 7900, 5700, and 6300 in NSRH, JUMC, AURH, and MKRH, respectively. The client transfer referral system with a neonatal referral form is used in these hospitals through three tiers of health facilities. Annually, in all hospitals, about 5,200 neonates are admitted, and in the previous four years, in all hospitals, 3,934 perinatally asphyxiated neonates were admitted.

### Source population

The source populations were all neonates diagnosed with perinatal asphyxia and admitted to the NICU wards of the tertiary hospitals of the west Oromia region.

### Study population

All neonates diagnosed with perinatal asphyxia and admitted to the NICU of the Western Oromia tertiary hospitals from January 1, 2018, to December 31, 2021, were included in the study population.

### Inclusion and exclusion criteria

#### Inclusion criteria

All neonates in the study area had PNA diagnoses and were admitted in the NICU between January 1, 2018, and December 31, 2021.

#### Exclusion criteria

Records of neonates whose admission date, discharge date, and outcome were not recorded on the chart and home delivery were excluded from the study.

### Sample size determination

For the first objective, the sample size was calculated by using the single population proportion formula with consideration of the following statistical assumptions based on previous study [30]: a 7.85 proportion of deaths (p = 0.0785 and q = 0.9215), a 95% confidence level, and a 5% margin of error.

$${\rm{Sample}}\,{\rm{size}}\,{\rm{ = }}\,\frac{{{{\left( {{{\rm{Z}}_{{\rm{\alpha /2}}}}} \right)}^{\rm{2}}}{\rm{P}}\,\left( {{\rm{1 - P}}} \right)}}{{{{\rm{D}}^{\rm{2}}}}}$$

Where; ni = initial sample size

Z = 1.96 the corresponding Z-score for the 95% CI.

α = confidence interval (95%).

P = proportions of death, 0.0785.

Ni = ((1.96)2. (0.0785) (0.9215)) / (0.05)2 = 111.15 and by considering incomplete patient records, 10 of the initial sample size was added, and the final sample size was 123.

The sample size was also calculated by using the second objective to check the adequacy of the sample size used for a survival sample size calculation power approach using STATA 14 software with Cox proportional assumptions. The four predictive variables included were cord prolapse, pregnancy-induced hypertension, maternal iron deficiency anemia, and convulsions from the previous study conducted in southern Ethiopia [30]. By using the Schoenfeld formula (Schoenfeld DA, sample-size formula for the proportional-hazards regression model, biometrics 1983;39 (2): 499–503.) [31].

$$E = \frac{{\left( {Z\frac{\alpha }{2} + Z\beta } \right)2}}{{p1p2\left( {\ln HR} \right)2}},\,\,\,\,\,\,\,\,PEV = 1 - \left( {\pi 1S1(T)} \right)\pi 2S2(T),N = \frac{E}{{Pev}}$$

Where E = number of events.

N = number of sample sizes.

P1 = proportion of the event among exposed.

Prev. = Probability of an event.

P2 = proportion of event among non-exposed.

From those predictors, the sample size obtained from maternal iron deficiency anemia was considered the final sample size of the study because it gives a maximum sample size. Using the probability of events (7.85), and a crude hazard ratio of (2.28), proportional withdrawals (10%), 95% level of confidence & and power of (80%); the final sample size becomes 655 (Table 1).

**Table 1:** Summary of sample size calculation to access incidence and predictors of mortality among neonates with PNA admitted at West Oromia Tertiary hospitals Ethiopia, 2022

Variables	Assumptions	CHR	Probability of events	Total sample size	Reference
Cord prolapse	Two side 95% CI	13.68	7.85	65	(34)
Pregnancy induced hypertension	Power = 80%	3.23		324	
Maternal Iron deficiency anemia	Ratio = 1:1	**2.28**		**655**	
Convulsion		3.27		317	

### Sampling technique and procedure

A sample of 655 charts of neonates with PNA was selected from 3,934 neonates’ charts admitted in the same case, and the subsequent unique chart numbers from the registration file were extracted using a computer-generated random number approach. Estimated previous four-year PNA neonates admitted to these hospitals were: Mettu Karl Referral Hospital (MKRH) (762), Jimma University Medical Centre (JUMC) (898), Wollega University Referral Hospital (WURH) (558), Nekemte Specialized Hospital (NSH) (970), and Ambo University Referral Hospital (AURH) (746) between January 1, 2018, and December 31, 2021.

**Fig. 1:**
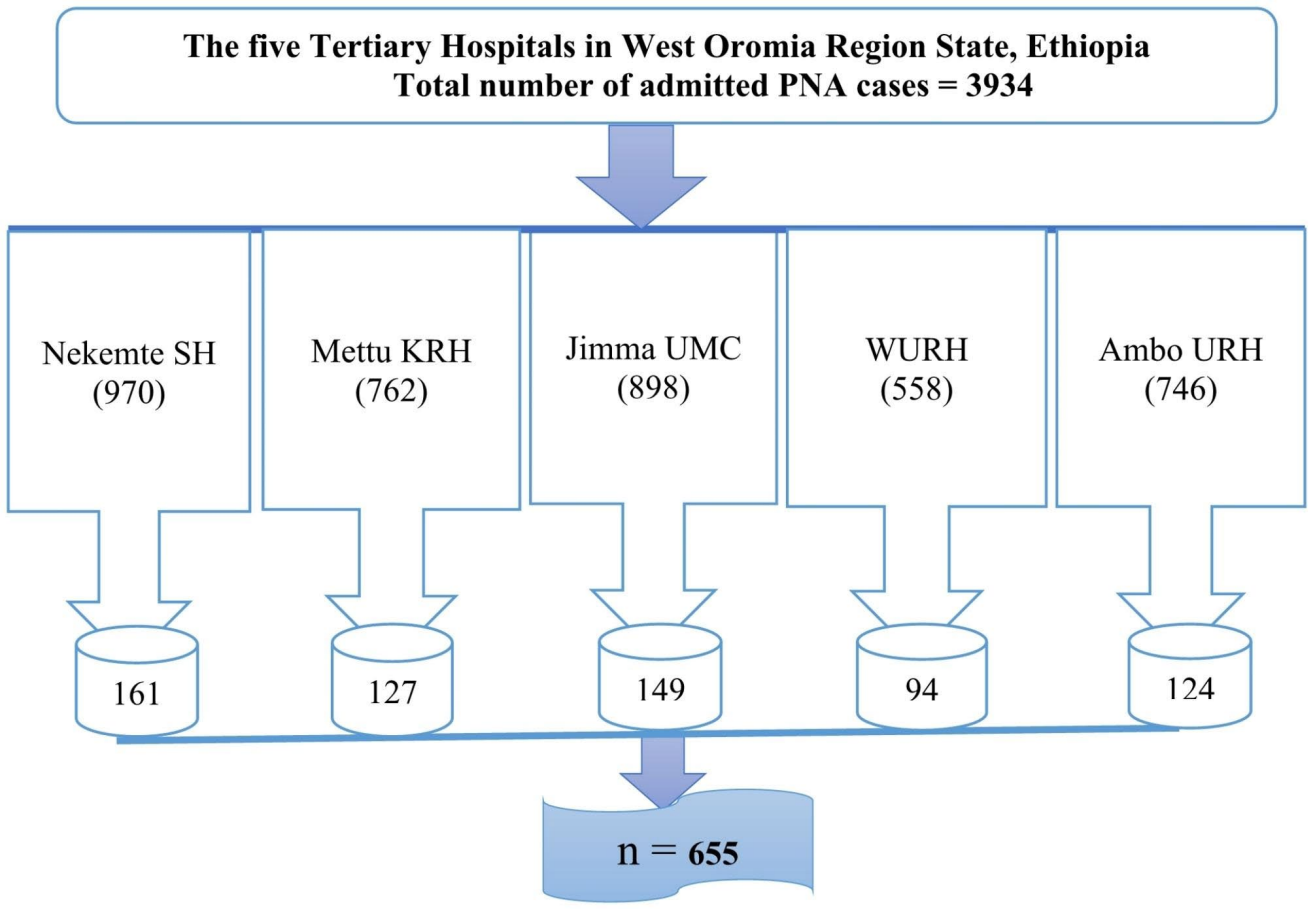
Schematic presentation of sampling procedure for incidence and predictors of mortality among neonates with perinatal asphyxia admitted at West Oromia tertiary hospitals from January 1, 2018 to December 31, 2021

Since the final sample size was (655), proportional allocation was done for each hospital. The sampling frame was prepared by collecting the identification numbers of PNA patients from the registration book. After identifying the patients who fulfilled the inclusion criteria, study subjects were selected by a simple random sampling technique using computer-generated random numbers (Fig. 1).

### Variables of the study

#### Dependent variable

Incidence of mortality from perinatal asphyxia.

#### Independent variables

**Sociodemographic factors**: age of mother, residence, distance from HF, neonatal age, and sex. **Maternal and obstetrical factors**: Pregnancy induced hypertension (PIH), premature rupture of membrane (PROM), mode of delivery, parity, gravid, meconium stained amniotic fluid (MSAF), antenatal care (ANC) visits, maternal Anemia, diabetic mellitus (DM), antepartum hemorrhage (APH), time of delivery, duration of labor, prolonged rupture of membrane, mode of delivery, cord prolapse, place of delivery, and presentation.

##### Neonatal and other comorbidities-related factors

APGAR score, congenital anomaly, hypoglycemia, hyperbilirubinemia, necrotizing enterocolitis (NEC), acute kidney injury, thrombocytopenia, gestational age, seizure, birth weight, birth type, weight for gestational age (WFGA), stages of hypoxic ischemic encephalopathy (HIE), hypocalcemia, cry at birth, and sepsis.

##### Medical care-related factors

antibiotics, oxygen, calcium gluconate, anti-epileptics, and time of initiation of enteral feeding.

### Operational definitions

#### Perinatal asphyxia

is considered when the fifth-minute APGAR score is less than seven [1, 2].

#### Event (death)

neonate died in the hospital, and the death summary was written on a chart after being diagnosed as PNA and admitted to the NICU.

#### Censored

neonates with PNA who did not develop the outcome of interest (death) until the end of the follow-up period or lost to follow-up, recovered from illness, discharged against medical advice, or transferred out to other health institutions without knowing the outcome.

#### Survival time

the time in days from admission to the development of the outcome variable (death) within 28 days of follow-up time.

#### Defaulted

PNA cases that are signed (by parents on behalf of their child) against treatment to leave treatment before the cure are completed.

#### Stages of HIE

were determined based on saranat’s classifications of the clinical diagnosis made by a health care provider (stage I, stage II, and stage III) [2].

### Data collection tool and procedure

A data extraction format was adapted from the peer-reviewed articles [16, 32, 33]. Data abstraction was designed based on study objectives and contains four parts: sociodemographic factors, maternal, and obstetric-related factors, neonatal and comorbidity factors, and medical care-related factors, which were obtained from medical records. The study used secondary data routinely recorded from patients’ charts. Two supervisors for supervision during data collection and five nurses working in the NICU were assigned as data collectors, and data were extracted by follow-up reviewing charts of patients. The NICU registration book is used to obtain a medical record number to identify individual patient cards. Then sociodemographic and other clinical data like date of admission, discharge, and a clear death summary were collected, with other neonatal and maternal clinical profiles collected accordingly.

### Data quality control assurance

To ensure the quality of the data, the data extraction tool was checked for the existence of variables in the registration format on the patient’s chart via a preliminary chart follow-up review of 5% (33 charts) of the sample, and the tool validity was checked by experts (a pediatrician and a neonatal nurse). Data collectors were nurses who had experience working in the NICU ward. In addition, data collectors were trained for one day at each hospital before data collection started. The data retrieval process was closely monitored by the principal investigator and/or two supervisors through a well-prepared and restricted data collection tool by Kobo Toolbox software.

### Data processing and analysis

Data inconsistency, coding errors, completeness, clarity, missing values, and data cleaning were checked before being exported. The data were exported to the STATA 14 version for further analysis.

Descriptive statistics of numeric variables were carried out, and categorical variables were presented using frequency, tables, and percentages. The incidence rate of mortality was also calculated by dividing the total number of new occurrences of death among PNA by the total number of person-days of follow-up. The Kaplan-Meier curve was used to estimate survival time. The log-rank test was used to compare survival experiences between independent groups. The proportional hazard assumption was checked both graphically and using a Schoenfeld residual test, which assesses the relationship between the scaled Schoenfeld residuals and time. Multicollinearity was checked between independent variables by a variance inflation factor. The frailty model was taken into account to handle variations among hospitals (Table 2).

**Table 2:** The frailty test procedure to handle variations between hospitals, among neonates with PNA, at West Oromia Tertiary Hospitals Ethiopia, 2022

Variables	Hazard Ratio	Std. Err.	z	P > z	[95% Conf. Interval]
Onset of labor	0.7647763	0.1312621	-1.56	0.118	0.5463096 1.070607
Preeclampsia	1.247635	0.2131684	1.29	0.195	0.8925925 1.743903
Birth type	0.7028621	0.2663027	-0.93	0.352	0.3344754 1.476985
Cry at birth	0.7945283	0.1721697	-1.06	0.288	0.5195878 1.214954
Stage of HIE	1.590064	0.2060773	3.58	0.000	1.233378 2.049902
Resuscitation	1.109238	0.3749334	0.31	0.759	0.5718869 2.15149
Thrombocytopenia	1.396109	0.2365395	1.97	0.049	1.001618 1.945971
Antibiotics	0.8541645	0.0794415	-1.69	0.090	0.7118289 1.024961
Antiepileptic	0.6149289	0.1042777	-2.87	0.004	0.441043 0.8573713
Congenital Anomaly	0.9260075	0.3593454	-0.20	0.843	0.4328109 1.981211
Revision of Orders	1.056392	0.0997418	0.58	0.561	0.8779241 1.27114
Duration of ROM	1.14914	0.1949888	0.82	0.413	0.8240229 1.602533
Duration of labour	1.050635	0.1756659	0.30	0.768	0.7570617 1.458051
APGAR at 5th min	0.7265933	0.1608552	-1.44	0.149	0.4708147 1.121328
Oxygen	1.897949	0.2969412	4.10	0.000	1.396724 2.57904
Distance of residence	1.891021	0.3186039	3.78	0.000	1.359202 2.630928
Newborn age at Admission	0.8334664	0.0908656	-1.67	0.095	0.6731145 1.032018
Time of delivery	1.051707	0.1544189	0.34	0.731	0.7887061 1.402407
_cons	0.0108179	0.0151348	-3.24	0.001	0.0006971 0.1678867
/ln_p	0.5310748	0.0543392	9.77	0.000	0.4245718 0.6375777
/ln_the	-15.12849	840.7158	-0.02	0.986	-1662.901 1632.644
p	1.700759	0.092418			1.528936 1.891893
1/p	0.5879727	0.03195			0.5285712 0.6540498
theta	2.69e-07	0.0002262			0.
LR test of theta = 0: chibar2(01) = 0.00, Prob > = chibar2 = 1.000					

The log-likelihood and Akaike Information Criteria (AIC) were applied to select the best-fitted model, and a model with a minimum AIC value was considered the best-fitted model. The goodness of fit of the model was assessed by Cox-Snell residuals and the Nelson-Aalen cumulative hazard function graph. A variable with a P < 0.2 in the bivariable analyses was considered a potential candidate variable for the multivariable analysis. A variable with a p-value ≤ 0.05 in the multivariable model was declared a significant predictor of the outcome of interest. Finally, the crude and adjusted hazard ratio (HR) with a 95% confidence interval (CI) was computed.

## Results

### Sociodemographic characteristics

Almost half of the mothers (326, or 52.925%) came from rural areas and came from an area with a distance of > 10 km (149, or 24.19%), and (464, or 75.32%) were aged between 21 and 34 years, and the median age of mothers was 24 years (IQR: 7). About (358, or 58.12%) of the neonates were male, and (270, or 43.83%) of them were admitted after 60 min of age. Most of the mothers (476, or 77.27%) were given birth in hospital (Table 3).

**Table 3:** Socio-demographic characteristics of the mother and neonate with perinatal asphyxia at West Oromia Tertiary Hospitals, Ethiopia, 2022

Variables	Category	Frequency	Percent
Maternal age	<=20 years	138	22.40
	21–34 years	464	75.32
	> 34 years	14	2.27
Residence	Rural	326	52.92
	Urban	290	47.08
Distance from HF	< 10 KM	467	75.81
	> 10 KM	149	24.19
Neonatal age at admission	< 30 min	236	38.31
	30–60 min	110	17.86
	> 60 min	270	43.83
Sex of the Neonate	Male	358	58.12
	Female	258	41.88
Place of delivery	Health center	140	22.73
	Hospital	476	77.27

### Maternal and obstetric-related factors

Half of the mothers (314, or 50.97%) attended the ANC three times or more during their current pregnancy. More than half of the mothers (365, or 59.25%) were multi-gravida, and about two-thirds (474, or 76.95%) of them had no history of pregnancy-induced hypertension. About (357, or 57.95%) of mothers had a history of non-prolonged rupture of membranes before delivery; more than half of them (366, or 59.42%) had no history of obstructed labor, and the majority of mothers (436, or 70.78%) were in labor for less than 18 h. Approximately half of the mothers, 303 (49.19%), had spontaneous vaginal deliveries during the night (328, or 53.25%). During the current pregnancy, the majority of the mothers (477, or 77.44%), (569, or 92.37%), (494, or 80.19%), and (604, or 98.05%) had no history of anemia, DM, abortion, or HIV infection, respectively (Table 4).

**Table 4:** Maternal and obstetric related characteristics of new-born with perinatal asphyxia at, West Oromia Tertiary Hospitals Ethiopia, 2022

Variables	Category	Frequency	Percent
ANC visit	No any visit	31	5.03
	One times	75	12.18
	Two times	196	31.82
	Three and above	314	50.97
Gravida	Primigravida	251	40.75
	Multigravida	365	59.25
Cord prolapse	Yes	61	9.90
	No	555	90.10
Presentation	Vertex	470	76.30
	Non vertex	146	23.70
PROM	Yes	480	77.92
	No	136	22.08
APH	Yes	59	9.58
	No	557	90.42
Amniotic fluid	Clear	222	36.04
	Meconium	394	63.96
Duration of ROM	< 18 h	357	57.95
	> 18 h	259	42.05
Duration of labor	< 18 h	436	70.78
	> 18 h	180	29.22
**Time of delivery**	Day time	288	46.75
	Night time	328	53.25
Obstructed labor	Yes	366	59.42
	No	250	40.58
Onset of labor	Induced	138	22.40
	Spontaneous	478	77.60
Mode of delivery	CS	182	29.55
	Instrumental delivery	131	21.27
	SVD	303	49.19
Pregnancy induced HTN	Yes	142	23.05
	No	474	76.95
Maternal Rh factor	Positive	584	94.81
	Negative	32	5.19
HIV status	Positive	12	1.95
	Negative	604	98.05
Hepatitis BV status	Positive	10	1.62
	Negative	606	98.38
VDRL	Positive	28	4.55
	Negative	588	95.45
History of DM	Yes	47	7.63
	No	569	92.37
Maternal anemia	Yes	139	22.56
	No	477	77.44

### Neonatal factors and other comorbidities

Most of the neonates (513, or 83.28%) had normal birth weight. About (488, or 79.22%), and (485, or 78.73%) of the neonates were moderately asphyxiated within the first and fifth minutes after birth, respectively, and half (308 or 50%) of them developed stage I HIE. The majority of the participants (521, or 84.58%) were born at term as singletons (602, or 97.73%). More than (566, or 91.88%) neonates were resuscitated, and about (394, or 63.96%) had no history of crying at birth. Generally, the majority of neonates (437, or 70.94%), (527, or 85.55%), (409, or 66.40%), (480, or 77.92%), (419, or 68.02%), and (492, or 79.87%) did not develop hyperbilirubinemia, NEC, AKI, thrombocytopenia, seizure, or RD, respectively (Table 5).

**Table 5:** Clinical characteristics of the new-born with perinatal asphyxia at, West Oromia Tertiary Hospitals Ethiopia, 2022

Variables	Category	Frequency	Percent
Birth weight	< 2500gm	85	13.80
	2500-3900gm	513	83.28
	>=4000gm	18	2.92
Gestational age	Preterm	95	15.42
	Term	521	84.58
Weight for GA	AGA	575	93.34
	SGA	12	1.95
	LGA	29	4.71
Birth type	Singleton	602	97.73
	Multiple	14	2.27
APGAR score at first minute	Moderate	488	79.22
	Severe	128	20.78
APGAR score at fifth minute	Severe	18	2.92
	Moderate	485	78.73
	Mild	113	18.34
History of cry at birth	Yes	222	36.04
	No	394	63.96
Stage of HIE	Stage I	308	50.0
	Stage II	232	37.66
	Stage III	76	12.34
History of resuscitation	Yes	566	91.88
	No	50	8.12
Hypoglycemia	Yes	337	54.71
	No	279	45.29
Hyperbilirubinemia	Yes	179	29.06
	No	437	70.94
NEC	Yes	89	14.45
	No	527	85.55
AKI	Yes	207	33.60
	No	409	66.40
Thrombocytopenia	Yes	136	22.08
	No	480	77.92
Seizure	Yes	197	31.98
	No	419	68.02
Sepsis	Yes	467	75.81
	No	149	24.19
Congenital anomalies	Yes	29	4.71
	No	587	95.29
Hypocalcemia	Yes	63	10.23
	No	553	89.77
Respiratory distress	Yes	124	20.13
	No	492	79.87

### Medical care-related factors

Two-thirds (387 or 62.82%) of the neonates’ orders were revised every 12 h. About half (328 or 53.25%) and (329 or 53.41%) of the asphyxiated neonates were treated with CPAP and initiated for enteral feeding after 48 h, respectively. The majority of the participants (471 or 76.46%) were given antibiotics, and more than one-third (197 or 31.98%) of the neonates were given anti-epileptic drugs (Table 6).

**Table 6:** Medical care related factors of the new-born with perinatal asphyxia at West Oromia Tertiary Hospitals Ethiopia, 2022

Variables	Category	Frequency	Percent
Type of oxygen therapy	CPAP	328	53.25
	Direct oxygen	288	46.75
Fluid therapy	Two third of total fluid	532	86.36
	Full total fluid	84	13.64
Antibiotics	Yes	471	76.46
	No	145	23.54
Calcium gluconate	Yes	487	79.06
	No	129	20.94
Anti-epileptic	Yes	197	31.98
	No	419	68.02
Phototherapy	Yes	153	24.84
	No	463	75.16
Revision of orders by physician	Every 6 h	115	18.67
	Every 12 h	387	62.82
	Every 24 h	114	18.51

### Overall neonatal outcome

**Fig. 2:**
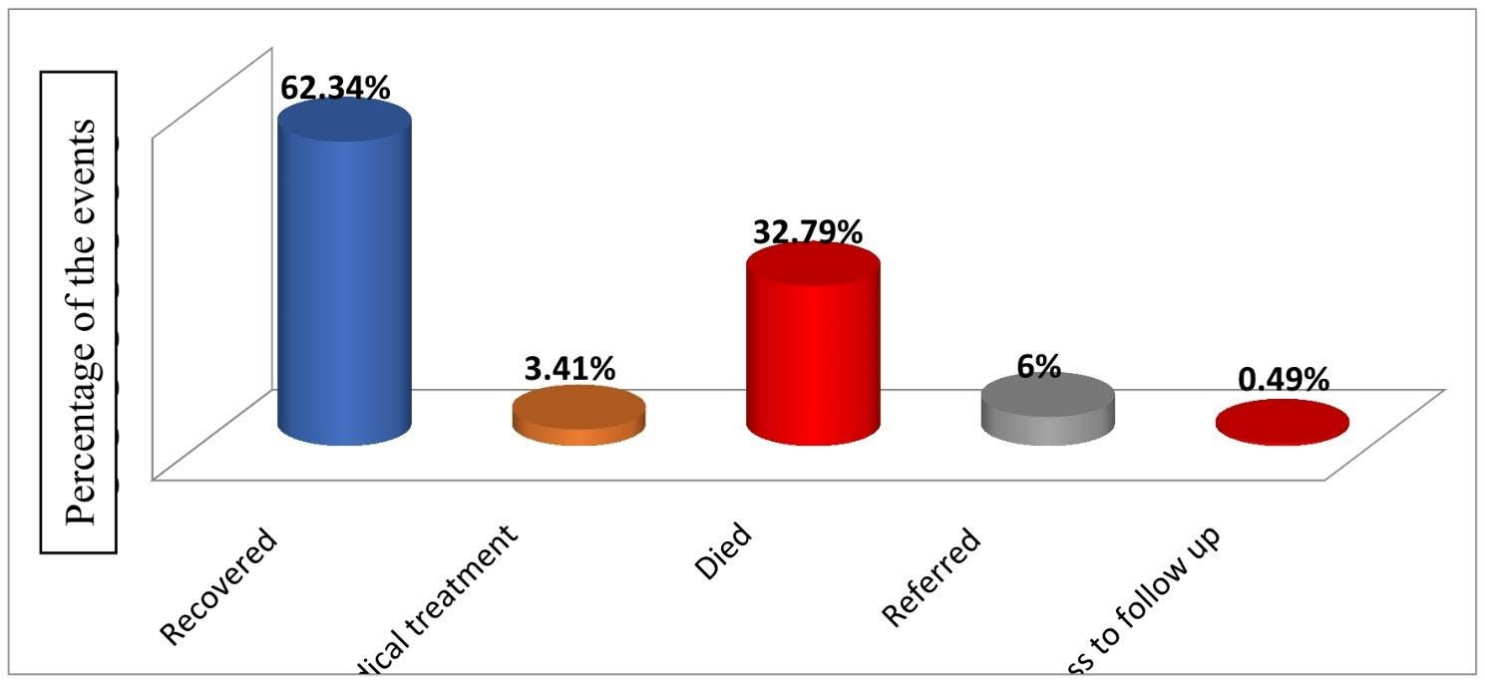
Overall outcome of neonates with perinatal asphyxia at West Oromia Tertiary Hospitals Ethiopia, 2022

From the total 28 days followed, 616 neonates (202, or 32.79%) developed the outcome variable (death) after being diagnosed with perinatal asphyxia and admitted to the NICU (Fig. 2).

#### Accessing proportional hazard assumption

Accessing the proportional hazard assumption was tested by using Schoenfeld residual proportional hazard assumption, and all the covariates fulfilled the assumption, and the overall global model result is described (Table 7).

**Table 7:** Global test for proportional hazard assumption using schoenfeld residuals for neonates with perinatal asphyxia, West Oromia Tertiary Hospitals Ethiopia 2022

Covariates	Rho	chi2	df	Prob > chi2
Onset of labour	0.02827	0.17	1	0.6789
Birth type	0.06635	0.90	1	0.3435
Obstructed labour	0.07193	1.12	1	0.2892
Preeclampsia	0.08947	1.69	1	0.1935
Cry at birth	-0.09199	1.62	1	0.2031
Stages of HIE	-0.04305	0.38	1	0.5364
Resuscitation	-0.06998	1.00	1	0.3173
Thrombocytopenia	0.06399	1.01	1	0.3158
Seizure	-0.02303	0.14	1	0.7053
Congenital Anomaly	0.00836	0.02	1	0.9017
Antibiotics	-0.06570	0.89	1	0.3444
Antiepileptic	0.00159	0.00	1	0.9791
Duration of ROM	-0.00533	0.01	1	0.9363
Duration of labour	-0.06003	0.78	1	0.3762
APGAR 1st minute	-0.02610	0.15	1	0.7029
APGAR 5th minute	-0.05174	0.57	1	0.4506
Acute kidney injury	-0.05224	0.58	1	0.4464
Oxygen	0.01157	0.03	1	0.8642
Distance from HF	0.05822	0.72	1	0.3978
Order revision	0.05783	0.84	1	0.3607
**Global test**		19.44	31	**0.9011**

All authors have read and approved the tables

### Model comparison

**Fig. 3:**
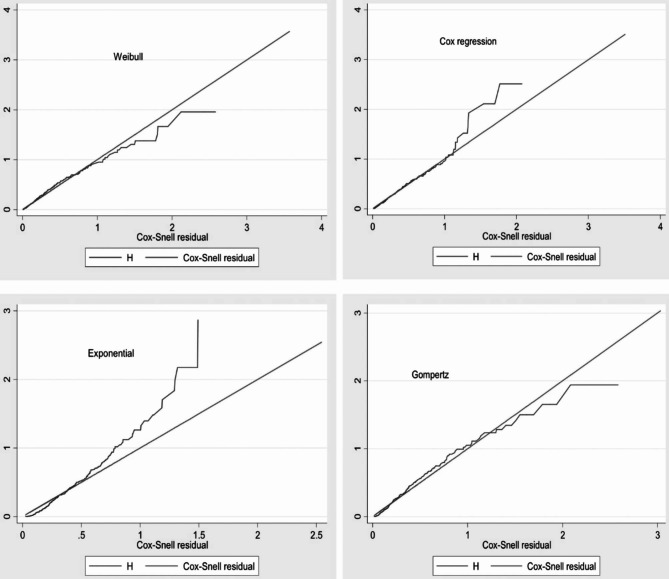
Summary of model comparison using Cox-Snell residual graph for neonates with perinatal asphyxia, West Oromia Tertiary Hospitals Ethiopia 2022 A residual is the difference between an observed data point and a predicted or fitted value and as the following graph indicates the Weibull regression fits than other models with hazards greatly followed the predicted hazard at 45’

Both semi-parametric and parametric proportional hazard models were fitted to determine the survival time to death and identify its determinants among neonates with perinatal asphyxia after the proportional hazard assumption was verified. The model comparison was graphically evaluated by Cox-Snell residual. Furthermore, model comparison was statistically examined using information criteria (AIC) and log-likelihood to determine which model was the best fit for the given data set. As a result, the Weibull regression model with an AIC of 895.47 was the model that suited the data (Table 8; Fig. 3).

**Table 8:** Summary of model comparison among the Cox proportional hazard model, parametric Cox- Regression models using AIC, among neonates with PNA, at West Oromia Tertiary Hospitals Ethiopia, 2022

Model	Baseline Hazard	Log-likelihood	AIC
Cox	Unspecific	-1113.198	2268.39
Weibull	Weibull	-423.7365	**895.47**
Gompertz	Gompertz	-442.4554	932.91
Exponential	Exponential	-459.1089	964.21

With a shape parameter (P) greater than zero, which indicates the hazard exponentially increases over time. The Weibull distribution was used to match the baseline hazard (the effect of time when all categorical variables are in a reference category) of experiencing an event or death (P = 1.71 95% CI: 1.53–1.90). The shared frailty was checked according to estimates, demonstrating that there is no difference between the hospitals in the distribution of unmeasured variables.

### Incidence of mortality

Of the total 616 neonates that were admitted with perinatal asphyxia (PNA), 202 (32.79%) (95% CI: 29.18–36.61) of them experienced the event of interest (death). The total neonate days observed over the full follow-up period were 5198 person-days, comprised of 883 from WURH, 1400 from NSH, 912 from MKSH, 1044 from JUMC, and 959 from AURH, respectively, with minimum and maximum follow-up times of 1 and 27 days. The 95% confidence interval for the median follow-up time was 14–20 days, or 15 days. Throughout the whole period of follow-up, the overall incidence of mortality was 38.86 per 1000 neonate (95% CI: 33.85–44.60). Regarding respective hospitals’ incidence of mortality, from WURH it was 30.57/1000 neonates (95% CI: 20.96–44.58), while 36.42/1000 neonates (95% CI: 27.68–47.93) from NSH, 41.66/1000 neonates (95% CI: 30.31–57.26) from MKSH, and 44.06/1000 neonates (95% CI: 33.00-58.82) from JUMC, and 41.71/1000 neonates (95% CI: 30.59–56.86) from AURH. The incidence of death at the beginning of the first 24 h, 3, 7, 14, and > 14 days was 17.85, 16.24, 11.85, 8.98, and 34.46 per 1000-neonates respectively.

## Overall survival function

**Fig. 4:**
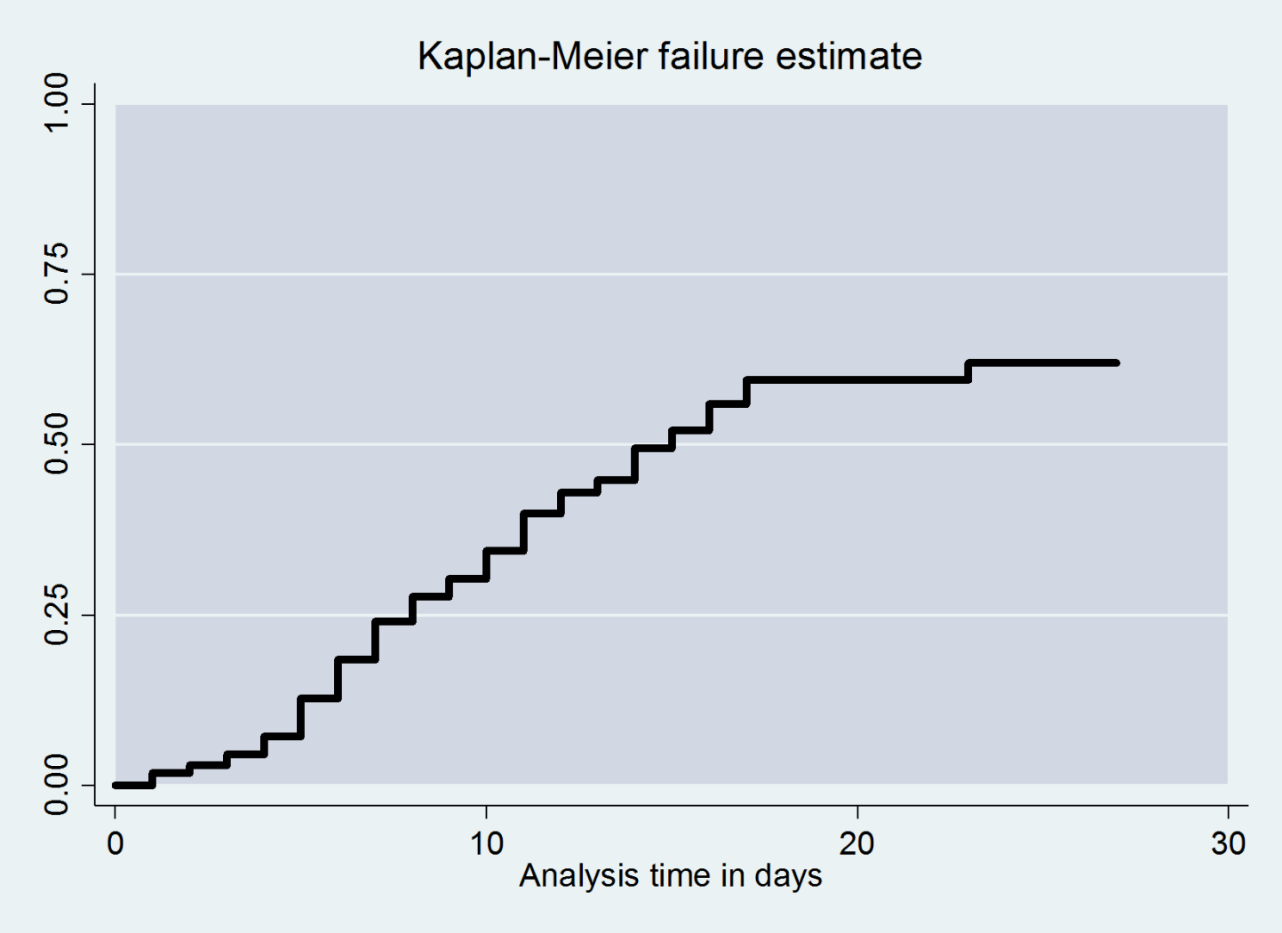
Kaplan Meier failure function of death among neonates with perinatal asphyxia at West Oromia Tertiary Hospitals Ethiopia, 2022

The overall Kaplan-Meier failure function demonstrated that neonates with PNA had a higher risk of dying during the follow-up period. On the first day after admission, there was a 1.78% chance of dying. The cumulative likelihood of dying at the end of 5, 10, and 15 days was 0.59 at five days, 0.57 at 10 days, and 0.50 at 15 days, respectively, and the least probability of death was observed at the end of 15 days of follow-up time (Fig. 4).

### Predictors of mortality among neonates with perinatal asphyxia

**Fig. 5:**
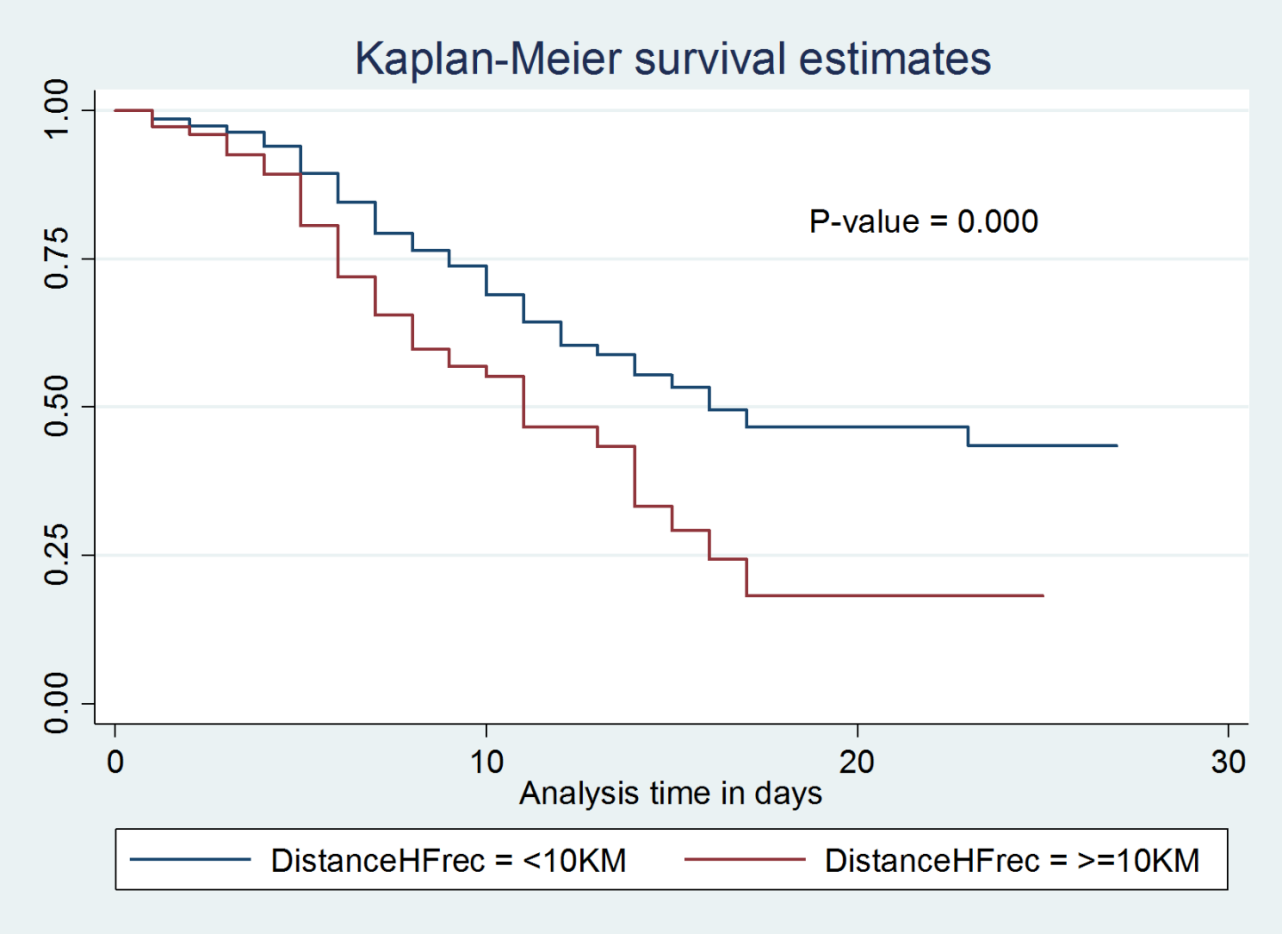
Kaplan-Meier inter-group comparison of survival function among neonates with PNA at West Oromia Tertiary Hospitals Ethiopia, 2022

**Fig. 6:**
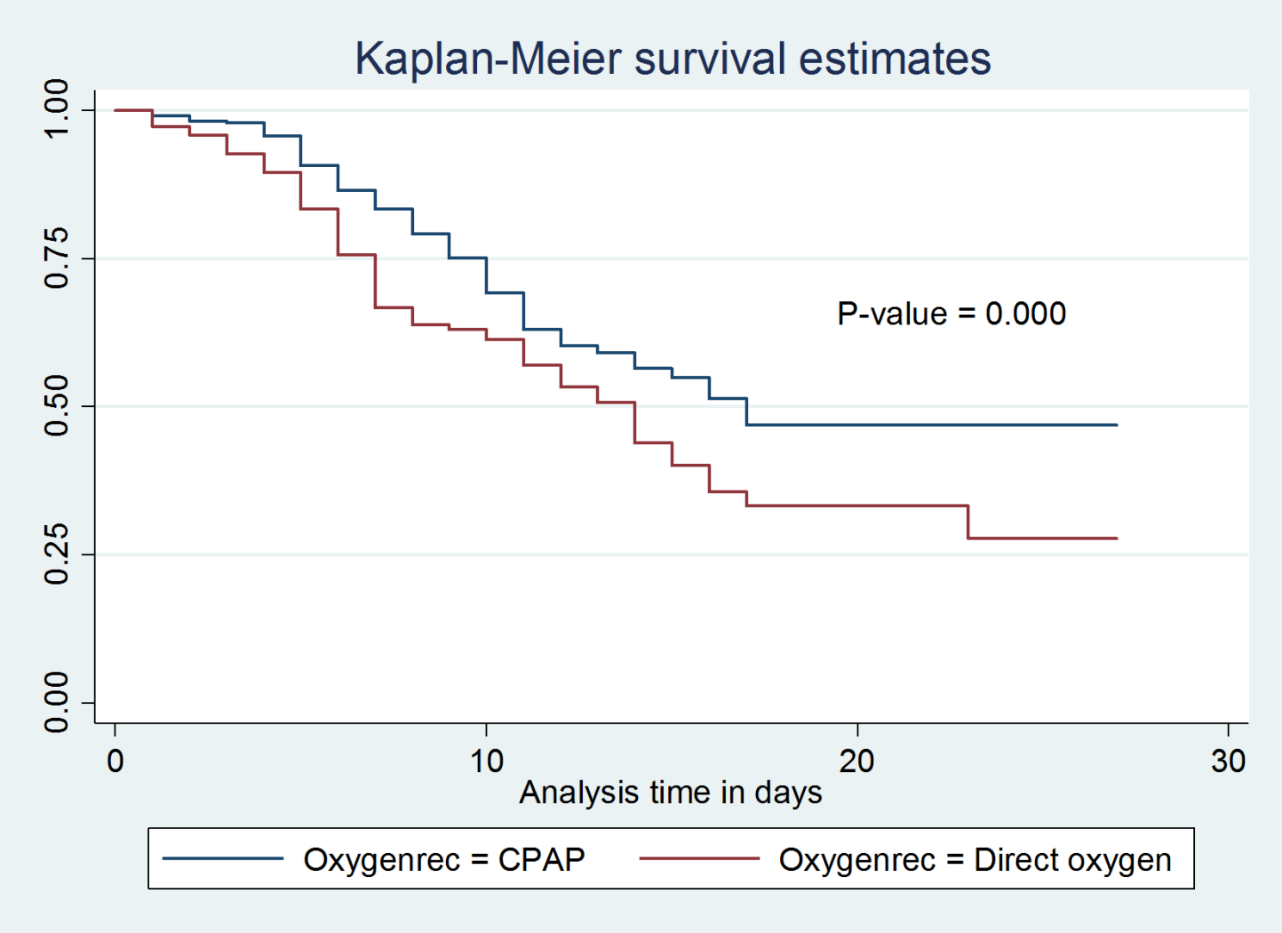
Kaplan-Meier inter-group comparison of survival function among neonates with PNA at West Oromia Tertiary Hospitals Ethiopia, 2022

With Kaplan-Meier and the log-rank test, the survival distribution for several categories of variables was tested for equality. In general, it was evident from the pattern that one group’s survivorship function was positioned above another group and that the lower curve had a higher chance of death than the upper curve (Figs. 5 and 6). The upper groups, on the other hand, as depicted by the Kaplan-Meier survival curve, had a high survival rate. Additionally, the log-rank test determined whether the observed difference was a statistical difference that could be seen on the KM graph.

In the current study, neonates whose families lived more than 10 km from a health facility had shorter survival times than their counterparts. Moreover, neonates whose mothers had a history of prolonged membrane rupture during their current pregnancy had a lower survival probability than those whose mothers had not.

This study also revealed that neonates who had low APGAR scores (< 3) at the fifth minute of birth were more likely to die when compared with those with scores greater than three. Furthermore, asphyxiated neonates who were treated with CPAP had a better probability of survival compared with neonates that were treated with direct oxygen. Moreover, asphyxiated neonates who had been diagnosed with stage III hypoxic-ischemic encephalopathy had a lower survival probability compared with those diagnosed with stage I and stage II HIE. These differences were statistically significant with a p-value of < 0.05.

### Bivariable and multivariable Weibull regression analysis

In the bivariable Weibull proportional hazard model, onset of labor, stage of HIE, obstructed labor, history of cry at birth, frequency of revision of order, type of oxygen used, antibiotics, antiepileptics, thrombocytopenia, resuscitation, preeclampsia, birth type, duration of labor, congenital anomaly, history of prolonged ROM, neonatal age at admission, APGAR score at the fifth minute, and distance from the health facility were associated with the incidence of mortality among neonates admitted with PNA. However, in the multivariable analysis, distance from HF, APGAR score at the fifth minute, stage of HIE, type of oxygen used, and prolonged rupture of the membrane were significant predictors of mortality among neonates admitted with PNA (Table 9).

**Table 9:** Bivariate and multivariate Weibull regression analysis for predictors of death among neonates with PNA, at West Oromia Tertiary Hospitals Ethiopia, 2022

Variables	Category	Status				CHR(95%CI)	AHR (95%CI)
		**Event**		**Censored**			
Stages of HIE	Stage I	74	24.02%	234	75.98%	1	1
	Stage II	85	36.64%	147	63.36%	1.04(0.76–1.42)	1.04(0.66–1.62)
	Stage III	43	56.58%	33	43.42%	2.06(1.41–3.01)	2.02 (1.18–3.47)*
Cry at birth	Yes	51	46.89%	171	53.11%	1	1
	No	151	38.32%	243	61.68%	1.38(1.00-1.90)	1.34(0.87–2.08)
Preeclampsia	Yes	51	35.91%	91	64.09%	1.28(0.87–1.65)	1.27(0.90–1.78)
	No	151	31.86%	323	68.14%	1	1
Birth type	Singleton	194	32.22%	408	67.78%	1	1
	Multiple	8	57.14%	6	42.86%	1.35(0.66–2.75)	1.47(0.69–3.10)
Onset of labor	Spontaneous	152	31.79%	326	68.21%	1	1
	Induced	50	36.24%	88	63.76%	1.38(1.00-1.91)	1.35(0.96–1.89)
Resuscitation at birth	Yes	11	39.11%	39	60.89%	1.42(0.77–2.60)	0.96(0.49–1.86)
	No	191	33.74%	375	66.26%	1	1
Thrombocytopenia	Yes	53	38.97%	83	61.03%	1.16(0.85–1.59)	1.26(0.89–1.78)
	No	149	31.05%	331	68.95%	1	1
Congenital anomaly	Yes	8	27.58%	21	72.42%	0.62(0.30–1.27)	0.62(0.29–1.32)
	No	194	33.05%	393	66.95%	1	1
Obstructed labor	Yes	89	35.60%	161	64.40%	1.22(0.92–1.61)	1.08(0.80–1.46)
	No	113	30.87%	253	69.13%	1	1
Duration of ROM	< 18 h	114	31.94%	243	68.06%	1	1
	> 18 h	88	33.97%	171	66.03%	1.37(1.04–1.81)	1.41(1.02–1.94)*
Antibiotics	No	38	26.20%	107	73.80%	0.72(0.51–1.03)	0.78(0.54–1.14)
	Yes	164	34.82%	307	65.18%	1	1
Antiepileptic	Yes	65	33.00%	132	67.00%	1	1
	No	137	32.69%	282	67.31%	1.27(0.94–1.71)	1.40(0.97–2.01)
Type of oxygen	Direct 02	108	37.50%	180	62.50%	1.61(1.22–2.13)	1.83(1.35–2.48)**
	CPAP	94	28.65%	234	71.35%	1	1
APGAR score at five minute	Mild	24	21.24%	89	78.76%	1	1
	Moderate	170	35.06%	315	64.94%	1.41(0.92–2.17)	1.28(0.79–2.08)
	Severe	8	44.44%	10	55.56%	1.76(0.79–3.92)	2.63(1.03–6.73)*
Duration of labor	< 18 h	136	31.20%	300	68.80%	1	1
	> 18 h	66	36.67%	114	63.33%	1.10(0.82–1.48)	0.91(0.65–1.26)
Distance from HF	< 10 KM	137	29.33%	330	70.67%	1	1
	> 10 KM	65	43.62%	84	56.38%	1.97(1.46–2.66)	2.25(1.60–3.17)**
Neonatal age at admission	< 30 min	148	31.62%	320	68.38%	1	1
	30 to 60 min	25	34.72%	47	65.28%	1.23(0.80–1.89)	0.98(0.62–1.55)
	> 60 min	29	38.16%	47	61.84%	1.01(0.68–1.51)	0.65(0.41–1.03)
Revision of order	Every 6 h	36	31.30%	79	68.70%	1	1
	Every 12 h	110	28.42%	277	71.58%	1.00(0.68–1.45)	0.94(0.63–1.41)
	Every 24 h	56	49.12%	58	50.88%	1.60(1.05–2.45)	1.25(0.75–2.07)

APGAR = appearance, pulse, grimace, activity, and respiration, CPAP = Continuous positive air pressure, HF = health facility, HIE = hypoxic ischemic encephalopathy, KM = kilometer, PROM = Premature rupture of membrane,

NB: ** significant (p-value < 0.001), * significant (p < 0.05).

Keeping other variables constant, the hazard of mortality was about 2 fold higher in asphyxiated neonates with stage III HIE (AHR: 2.02; 95% CI: 1.18–3.47) than in stage I and stage II HIE. Neonates whose mothers had a history of prolonged membrane rupture had a 1.41-fold higher hazard of death than those whose mothers had not (AHR: 1.41; 95% CI: 1.02–1.94). Neonates with an APGAR score of less than 3 at the fifth minute were about 3 times more likely to die (AHR: 2.63; 95% CI: 1.03–6.73) than neonates with an APGAR score of greater than 3 at the fifth minute by keeping out other variables constant. Moreover, neonates who traveled more than 10 km from HF had a 2-fold hazard of death than those who traveled less than 10 km (AHR: 2.25; 95% CI: 1.60–3.17) while other variables remained constant. Furthermore, when all other variables were held constant, neonates on direct oxygen had about a 2-fold higher hazard of death (AHR: 1.83; 95% CI: 1.35–2.48) than asphyxiated neonates on CPAP.

## Discussion

The primary objective of this study was to estimate the incidence rate of death and assess its predictors among asphyxiated neonates admitted to the NICU. From the total of 616 asphyxiated babies enrolled in this study, 202 (32.79%) (95% CI: 29.18–36.61) neonates experienced the event of interest (died). Throughout the full follow-up period, the overall incidence of death was 38.86 per 1000 neonates (95% CI: 33.85–44.60).

This finding is lower than that of the study conducted in northwest Ethiopia, which was (53.49 per 1000) [33]. This marked difference might be due to the study area, as the current study is multi-intuitional, while the northwest Ethiopia study was conducted monocentrically as a single hospital incidence represents a limited area of the population. Another possible justification could be due to the difference in overall included data, as this study followed data for four years, while the northwest Ethiopia study followed data three years. Meanwhile, different from the period of data incorporation, neonatal care is improving in the current period, as this study used more recent data than the northwest Ethiopia study [33].

According to this study’s findings the incidence of mortality is higher than the study conducted in Brazil 1.71 deaths per 1,000 [34]. This great difference might be due to the difference in study methods, as this study used a prospective cohort while our study used a retrospective follow-up because the prospective follow-up study follows the actual patients up to the occurrence of the event. Another reason could be due to differences in geographical area, health facilities relative to the total population, the skill of neonatal resuscitation among midwives and nurses, the structure of the NICU, and the advanced technology used in neonatal care [34].

The proportion of deaths in this finding is greater than in the studies conducted in southern Ethiopia (7.85%) [30], Addis Ababa (24.09%) [32], Nigeria (18%) [23], and Tanzania (23%) [7]. The difference between the current study findings and the study of southern Ethiopia might be due to the difference in study design, as this study used a retrospective follow-up and the study of southern Ethiopia was a prospective follow-up. In addition, it might be due to differences in health care provider level; in Addis Ababa, even if it is not adequate, there are neonatologists, but not in our study area, which may make a difference in neonatal care [30].

The variability of results obtained in this study and a study conducted in Addis Ababa might be due to the discrepancy in sample size, the discrepancy in health facilities and health professionals in these study areas, and political instability in the current study area. In other words, the study conducted in Addis Ababa did not consider the comorbidities like sepsis, respiratory distress, and medical care that have been carried out for neonates, on which the current study was focused [32].

To justify the difference, the study conducted in Nigeria used clinical laboratory investigations as inclusion criteria, but in this study, clinical diagnosis was the only criterion for the diagnosis of PNA, and there might be differences in the study population and treatment modalities in two different countries [23]. Meanwhile, the reason why the study conducted in Tanzania was lower might be because the study conducted in Tanzania was monocenteric and followed two years of PNA data, while this study followed four years of multi-center data. In addition to the aforementioned factors, the variability could be explained by the fact that the Tanzanian study excluded neonates who died within the first 30 min of admission, whereas the current study included all types of deaths [7].

The proportion of deaths in the current study is lower than the studies conducted in northern Ethiopia (37.5%) [35], and eastern Ethiopia (55.3%) [28]. The difference could be attributed to the study period, as the northern Ethiopia study only looked at two years of data, whereas the current study looked at four years. Another reason might be that the northern Ethiopia study included all NICU-admitted neonates, whereas this study evaluated neonates with PNA. Meanwhile, there could be differences in sample size and study design [35].

The discrepancy between this study and the study conducted in eastern Ethiopia might be due to the monocenteric study and the difference in sample size. Meanwhile, in the Eastern Ethiopian study, all neonates admitted to the NICU were included, whereas in the current study, only perinatally asphyxiated neonates were evaluated [28].

In this study, severe asphyxia or less than 3 APGAR scores at the fifth minute is an independent predictor and increases the hazards of death 3 times among neonates with PNA. This is supported by the study conducted in Addis Ababa [36], Nigeria [37], Tanzania [7], and Cameroon [38]. The reason for this finding might be perinatal asphyxia, which is a lack of blood flow or gas exchange to or from the fetus in the period immediately before, during, or after the birth process. The APGAR score is a numeric assessment of the neonatal condition at birth and can be affected by numerous factors, including gestational age, medication taken by the mother, resuscitation efforts, and cardiac, respiratory, or neurological abnormalities in the baby. If the neonate is severely asphyxiated at the golden times of the first and fifth minutes, the tissues and vital organs (muscle, liver, heart, and ultimately the brain) will develop an oxygen deficit. Anaerobic glycolysis and lactic acidosis will result and also lead to death [1, 39]. Improving the resuscitation skills of NICU and labor and delivery health care providers, prompt referral to the hospital, and strict follow-up during labor and delivery are needed to alleviate the occurrence of low APGAR scores in the perinatal period.

In the current study, using direct oxygen rather than CPAP is another predictor of mortality and increases the hazard of death almost 2 times. This finding is supported by different studies conducted in Ugandan [40], and China [41]. Justification for this finding could be that CPAP is a simple and effective respiratory support modality used to support neonates with respiratory failure by providing constant pressure maintained throughout the respiratory cycle but with no additional inspiratory pressure support. CPAP keeps alveoli open, improves oxygenation by reducing the amount of blood shunted through atelectasis while the infant breathes spontaneously, and reduces the hazards of mortality from asphyxia [42]. As this predictive variable indicates, healthcare providers should be aware of the use of CPAP to minimize the incidence of death among PNA neonates.

A neonate who develops stage III HIE is an independent factor that predicts mortality and increases the hazards of death by two. This finding is supported by the study conducted in northwest Ethiopia [33], India [18, 43–45], Nigeria [23, 46], and Cameroon [38]. It could be due to the fact that, hypoxic ischemic brain injury is an ongoing process composed of several different phases. When the primary critical energy failure occurs, an uncontrolled release of excitatory neurotransmitters begins, starting the ischemic cascade that damages neuronal cells (both at the cytoplasmic and mitochondrial levels), disrupts the brain-blood barrier, and activates an important inflammatory response. These result in failure of oxidative metabolism, cytotoxic edema, and the accumulation of excitotoxins, which may lead the neonates to death [47]. Because these neonates had multi-organ failure, we need to pay special attention to them while providing cares to reduce mortality.

In this study, PNA neonates of mothers who had a history of PROM in their current pregnancy are another factor that predicts mortality among PNA and increases the hazards of death by about 1.41 times. This finding is supported by the studies conducted in India [48], and Cameroon [38]. The reasons could be due to the complications of prolonged rupture of the membrane (amnionitis and endometritis), which both put the fetus at a high risk of developing an overwhelming infection in the bloodstream (sepsis), which can exacerbate hypoxia and lead to death [2]. Minimizing the time of labor after rupture of the membrane could ameliorate this condition, which is a predictor of death among PNA.

According to the findings of this study, distance from a health facility is a significant predictor of death from PNA and doubles the hazards of death. This finding might be due to a delay in seeking health care, which can prolong the initiation of care for asphyxiated neonates, increase the severity of the problem, and lead to death. It is also supported by the study conducted in Uganda, as delay in seeking health care is directly associated with the distance from a health facility [49]. Providing improved community-based newborn care and providing health education for pregnant mothers on how to plan where to give birth and when to seek care can change the effect of distance from a health facility on PNA neonates’ ability to survive.

### Limitations of the study

The primary weakness of the study could be incomplete medical records because it was based on secondary data. In this regard, medically recorded data did not provide information on certain important factors, such as mothers’ educational attainment, nutritional status, and monthly income. Furthermore, because the study was conducted in a hospital, neonatal mortality may have been underestimated, as home deliveries that ended in neonatal deaths were not properly reported.

## Conclusion

The study found that neonatal mortality among perinatal asphyxia patients remains high. Besides, the overall incidence rate of mortality was 38.86 per 1000 neonates. The type of oxygen used, residence distance, history of prolonged ROM, and stages of HIE were significant predictors of mortality. Therefore, pregnant mothers should get information on when to seek care following their pregnancy. Providing appropriate follow-up for PNA neonates with stage III HIE, minimizing the time of labor after rupture of membrane, and evaluating the treatment options regarding the type of oxygen for PNA neonates are also mandatory to reduce mortality from perinatal asphyxia. Moreover, future researchers should conduct an interventional study to address further significant predictors of mortality in asphyxiated neonates.

## Data Availability

All data generated or analyzed during this study are included in this published article.

## Abbreviations and Acronyms

ANC: Antenatal Care
APH: Ante-partum Hemorrhage
AURH: Ambo University Tertiary Hospital
CPAP: Continuous Positive Air Pressure
EDHS: Ethiopian Demographic Health System
ETB: Ethiopian Birr
GA: Gestational Age
GDM: Gestational Diabetic Mellitus
HIE: Hypoxic Ischemic Encephalopathy
INO2: Intra, nasal Oxygen
IQR: Interquartile Range
JUMC: Jimma University Tertiary Hospital
MKRH: Karl Mettu Tertiary Hospital
MOD: Mode Of Delivery
MSAF: Meconium Stained Amniotic Fluid
NICU: Neonatal Intensive Care Unit
NSRH: Nekemte Specialized Tertiary Hospital
PI: Principal Investigator
PIH: Pregnancy Induced Hypertension
PNA: Perinatal Asphyxia
PPH: Postpartum Hemorrhage
PROM: Premature Rupture Of Membrane
UoG: University Of Gondar
VDRL: Venereal Disease Research Laboratory
WURH: Wollega University Referral Hospital
